# 

**Published:** 1969-04

**Authors:** 


### BRISTOL MEDICO-CHIRURGICAL JOURNAL

*(Incorporating the Medical Journal of the South-West)*

Established 1883

Vol. 84 (ii) April 1969 No. 310

### *Contents*

| | Page |
|---|---|
| THE CARE OF THE ELDERLY<br>*W. H. Lloyd; A. W. Macara; P. S. Sinclair* | 33 |
| COLDHARBOUR FARM—THE FIRST FIVE YEARS<br>*W. A. Heaton-Ward* | 46 |
| HOST RESISTANCE AND HOSPITAL INFECTION<br>*W. A. Gillespie* | 51 |
| THE CHANGING MEDICAL CURRICULUM | 56 |

***PUBLISHED QUARTERLY***

***ANNUAL SUBSCRIPTION* 16/- *POST FREE***

### BRISTOL MEDICO-CHIRURGICAL SOCIETY

President 1968-1969 : Mr. A. L. Eyre-Brook

President-elect : Dr. F. J. W. Lewis

Honorary Secretary : Dr. A. T. M. Roberts, 8 Harley Place, Bristol 8.

Honorary Treasurer : Dr. J. Macrae, Ham Green Hospital, Pill.

The Society was founded in 1874. In 1883 publication of this Journal began, and has continued quarterly since then. During the years 1949 to 1962 it appeared under the title of " The Medical Journal of the South West ".

The Society founded a medical library in 1890, which in 1892 was combined with that of the Bristol Medical School. This became the University of Bristol Medical Library in 1925, and an agreement in that year confirmed the privilege of Members to use the University Medical Library.

### THE BRISTOL MEDICO-CHIRURGICAL JOURNAL

*Editorial Committee*

*Editors :*

Dr. N. J. Brown, Southmead Hospital, Bristol
Dr. A. B. Raper, Royal Infirmary, Bristol

*Members :*

| | |
|---|---|
| Mr. H. B. Griffith | Mr. J. B. M. Roberts |
| Dr. M. B. Lennard | Professor R. C. Wofinden |
| Dr. A. E. Read | Dr. D. W. Wright |

*Secretary :*

Mr. A. E. S. Roberts, The Library, Medical School, University Walk, Bristol, 8.

The Hon. Treasurer and Hon. Secretary of the Society.

### NOTICE TO CONTRIBUTORS

The Journal is especially concerned to provide a place for the recording of medical thought, interests, and practice in and around Bristol. It therefore welcomes articles that, as well as being of immediate interest to members, will document the local and contemporary medical scene for our successors.

Original articles are invited provided that they have not been published elsewhere. References in the text should be by author's name, with year of publication in parenthesis; they should be listed at the end in alphabetical order. Illustrations should be supplied ready for reproduction. Line drawings should be in indian ink on firm white paper or board. The author will be informed if it is found necessary to ask him to pay for the making of blocks. The Editorial Committee reserves the right to make literary corrections.

The following contributions will be especially welcome: notes of forthcoming events, meetings held, degrees conferred, staff appointments, honours and public appointments and other local news.

Printed by

F. Bailey & Son, Dursley, Glos.

